# Expanding Conditions for Newborn Screening—Findings from an Untargeted Metabolomics and Lipidomics Discovery Study

**DOI:** 10.3390/ijns12040077

**Published:** 2026-09-24

**Authors:** Alex J. Ashenden, Tomas Rozek, Lucy Anastasi, Ayesha Chowdhury, Khoa Lam, Carol Wai-Kwan Siu, Jovanka King, Tracy Merlin, Nicholas Smith, Benjamin Saxon, Drago Bratkovic, Christopher Barnett, Hamish S. Scott, Enzo Ranieri, Jennie Louise, Karin S. Kassahn

**Affiliations:** 1 Department of Genetics and Molecular Pathology, SA Pathology, Adelaide, SA 5000, Australia; 2 South Australia Neonatal Screening Centre, SA Pathology at Women’s & Children’s Hospital, Adelaide, SA 5006, Australia; 3 School of Medicine, College of Health, Adelaide University, Adelaide, SA 5000, Australia; 4 Allergy & Clinical Immunology Department, Women’s and Children’s Hospital Network, Adelaide, SA 5006, Australia; 5 Immunology Directorate, SA Pathology, Adelaide, SA 5000, Australia; 6 Adelaide Health Technology Assessment (AHTA), School of Public Health, The University of Adelaide, Adelaide, SA 5000, Australia; 7 Neurology and Clinical Neurophysiology, Women’s and Children’s Health Network, Adelaide, SA 5006, Australia; 8 Haematology and Oncology Clinic, Women’s and Children’s Hospital, Adelaide, SA 5006, Australia; 9 Metabolic Clinic, Women’s and Children’s Hospital, Adelaide, SA 5006, Australia; 10 Paediatric and Reproductive Genetics Unit, Women’s and Children’s Hospital, Adelaide, SA 5006, Australia; 11 Centre for Cancer Biology, an Alliance Between SA Pathology and the University of South Australia, Adelaide, SA 5000, Australia; 12 Sydney Children’s Hospital at Westmead, Sydney, NSW 2145, Australia; 13 South Australian Health and Medical Research Institute, Adelaide, SA 5000, Australia

**Keywords:** newborn screening, early detection, metabolomics, lipidomics, multi-omic NBS, genomic newborn screening

## Abstract

Advances in metabolomics and genomics present new opportunities to expand newborn screening. Traditional screening approaches have relied on condition-specific biomarkers; however, untargeted metabolomic profiling may enable broader detection of genetic conditions. In this study, we developed and evaluated a novel metabolomic screening approach based on defining a reference profile representing a healthy newborn population. Retrospective dried blood spot samples from 2948 newborns were analysed using untargeted metabolomics and lipidomics, generating more than 30,000 features. A discovery cohort of over 2000 healthy newborns and newborns with genetic conditions was used to identify 1115 features that best discriminated between groups. These features formed the basis of a screening algorithm, which was subsequently evaluated in an independent validation cohort containing genetic conditions not represented in the discovery cohort. Across the discovery and validation cohorts, 180 cases representing 113 unique genetic conditions were assessed, including metabolic, haematological, neurological, and syndromic conditions. In the independent validation cohort, the algorithm achieved an area under the receiver operating characteristic curve of 0.799, with a specificity of over 99%. These findings demonstrate the potential of untargeted metabolomic profiling to identify newborns at increased risk of genetic disease and support future exploration in a multi-omic newborn screening design.

## 1. Introduction

Newborn bloodspot screening (NBS) programmes reduce childhood morbidity and mortality by enabling early detection and intervention for rare diseases. Worldwide these programmes have seen a high level of success and public confidence since their implementation in the 1960s. NBS began with a test detecting high levels of phenylalanine in presymptomatic patients with phenylketonuria (PKU) [1,2]. In Australia, NBS programmes have since expanded to include a broad range of other inborn errors of metabolism (IEM) as well as other genetic conditions such as cystic fibrosis (CF), spinal muscular atrophy (SMA) and severe combined immunodeficiency (SCID) [3].

Targeted metabolic testing is a fundamental component of most NBS programmes, whereby dried blood spot (DBS) cards and multiplexed targeted metabolic assays are used to screen for a variety of amino acid, organic acid, and acylcarnitine disorders at low cost and rapid turn-around time [4]. There are hundreds of other serious, but treatable conditions, which collectively affect 1 in 32 Australian newborns, for which early detection through NBS programmes could offer life-changing treatment options. There is thus great potential for metabolomics to be used to screen for a far wider range of conditions, if relevant sets of features could be identified. Prior knowledge of relevant metabolic pathways can inform biomarker discovery, as well as untargeted metabolomics in which thousands of analytes are measured to determine which may be useful for the diagnosis of a particular condition [5,6,7,8]. These approaches to biomarker discovery are, however, unfeasible at-scale; applying them to hundreds of conditions would require a large number of case samples for robust predictive modelling, as well as later validation, for each rare condition, when these conditions are individually rare or ultra rare [9]. Poor reproducibility, poor generalizability, and the potential need for new instrumentation or workflows are further considerations which may preclude implementation of such approaches at population scale. For these reasons, we explored here untargeted methods to screen for a range of genetic conditions concurrently with a single scalable test, without the need for targeted biomarker identification.

One approach is to attempt to find a relatively small subset of metabolomic features whose joint distribution in unaffected control samples could be used as a reference against which a range of conditions may be detected as outliers. Rather than finding specific biomarkers that are diagnostic of an individual condition, samples could be classified simply as ‘outliers’ with respect to a carefully created reference distribution of features. This would then prompt second-tier testing to confirm the abnormal screening result and identify any underlying condition. This second-tier testing could be performed using biochemical assays, as has mostly been the practice to date. However, with the advent of next-generation sequencing (NGS), genomic approaches now provide an attractive alternative [10].

In standard-of-care NBS (stNBS), genomic and molecular testing is currently used for only a few conditions, most notably SMA, SCID and second-tier testing for CF, and is typically performed using quantitative real-time PCR or targeted sequencing methods [11]. For diagnostic investigations, the increasing affordability of whole-genome sequencing (WGS) has made it an attractive approach for genomic testing. For application in newborn screening, WGS data can be pre-filtered to a virtual gene panel and reporting limited to variants considered to be pathogenic and clinically actionable in a newborn screening context [12]. Genomic newborn screening studies are making use of advances in WGS to perform large-cohort analyses in parallel with traditional NBS [13,14,15]. This approach can vastly increase the number of screened conditions; however, there are many other issues, including workforce, cost, data management, and ethical concerns that need to be addressed before genomic newborn screening could be implemented at population scale. By first screening newborns to identify those with outlier metabolomic profiles, WGS could be integrated as a second-tier testing option in a multi-omic pathway [16].

In this study, we report on the development and performance of a novel metabolomic screening algorithm. We used retrospective samples, untargeted metabolomic analyses of lipid and metabolite features and statistical approaches to create a normal reference distribution against which newborns with a wide range of genetic conditions were identified. We tested the performance of the algorithm using a validation cohort comprised of DBS from newborns with a different set of genetic conditions from those included in the discovery cohort (Figure 1). This retrospective study forms the basis for further studies as part of the NewbornsInSA project [17], which will explore the use of the screening model developed here in a prospectively recruited cohort and cross-reference with whole genome sequencing results to test its efficacy as a first-tier screening tool.

## 2. Materials and Methods

### 2.1. Establishing a Discovery and Validation Cohort

For this study, a discovery and validation cohort were created using newborn DBS cards collected from the South Australian population between 2019 and 2024. A preliminary study of the effect of sample age on metabolite stability revealed substantial storage age-related changes which were not consistent between metabolites (Supplementary Figure S1). To avoid potential confounding factors related to storage age, we therefore restricted analyses to samples collected no earlier than 2019. This study has been approved by the Women’s and Children’s Health Network Human Research Ethics Committee (2022/HRE00258).

In both the discovery and validation cohort, the inclusion criteria for control samples were: DBS collected after established feeding, two days after birth, in the year 2019 or later, baby was born in a South Australian hospital, at 39–42 weeks gestational age, birth weight between 2500–4000 g, and no recorded abnormal results from traditional NBS, no clinical notes at time of DBS collection, and no known genetic diagnoses identified. The last time of follow-up was between 0 and 4 years of age; genetic diagnoses made after this age or conditions that may only present later in childhood would have been unknown at the time of this analysis. Case samples were identified through searches of the SA Pathology NBS and genomic databases and through notification by clinical staff associated with this study. While we prioritised genetic conditions which have early onset in childhood and available treatment, we specifically included the 613 conditions screened for in the NewbornsInSA prospective study (target conditions, Supplementary Table S1), some other genetic conditions (non-target conditions), and carriers and stNBS positive samples without molecular diagnosis to assess the ability of the metabolomics methods to discriminate these cases (Supplementary Tables S2–S4). Case samples were further restricted to DBS cards with sufficient sample material and non-ambiguous molecular diagnoses. Case samples were not controlled for birth weight or gestational age. All samples used in this study were collected as part of routine/standard newborn screening at 48 h of age.

### 2.2. Sample Preparation and Internal Standards

All metabolomic and lipidomic studies were performed using DBS cards, which were retrieved from archives at the SA Neonatal Screening Centre, SA Pathology, Women’s and Children’s Hospital, South Australia. From each DBS, a 6 mm punch was placed into a 96-deep well filter plate (Agilent Captiva 0.45″ polypropylene). All plates included a matrix blank, internal standard matrix blank, two quality control blood spots from a pooled mix, a pure internal standard, and a solvent blank. Internal standards for metabolomics consisted of isotopically labelled amino acids, carnitines and organic acids used for NBS and metabolic studies (Supplementary Table S5a). Internal standards for lipidomics consisted of d7-cholesterol, C22-d3-docosanoic acid, d8-arachidonic acid, and a variety of isotopically labelled lipids (Supplementary Table S5b). Internal standards for lipidomics were made in-house to provide a similar combination to the commercial lipidomic analytical standard mixture SPLASH^TM^ II LIPIDOMIX^TM^ (Avanti Research, Alabaster, AL, USA).

It was expected that analytes commonly measured in current NBS programmes such as phenylalanine and carnitines would exhibit a higher area response factor in samples from cases with known diagnoses of PKU, medium-chain acyl-CoA dehydrogenase (MCAD) deficiency, and very long-chain acyl-CoA dehydrogenase (VLCAD) deficiency, for example. Analysis of these analytes was performed by comparing the response factor against surrogate internal standards. For preliminary analysis, samples in which the area ratio (analyte/internal standard) was greater than the 99.9th or 99.5th percentile of area ratio for all samples were considered to have a notably elevated area response factor.

### 2.3. Consumables

Liquid Chromatography-Mass Spectrometry (LC-MS) grade water, methanol, isopropanol, acetonitrile, and formic acid were purchased from Merck (Burlington, MA, USA). Ammonium formate was purchased from Fluka™. Ammonium formate eluent additive for LC-MS was purchased from Honeywell International Inc (Morristown, NJ, USA). Agilent Captiva 0.45″ polypropylene 96-well filter plates were purchased from Agilent Technologies Australia Pty Ltd. (Mulgrave, VIC, Australia).

### 2.4. Extraction Procedure for Metabolomic and Lipidomic Analysis

For metabolomics, a 50 µL internal standard custom mix (Supplementary Table S5a) was added to samples along with 400 µL of methanol-water extraction solution (90:10 *v*/*v*). After shaking for 30 min, samples were filtered under vacuum into a 96-deep well plate (Agilent, 1 mL depth). Filtered samples were dried under nitrogen and re-constituted for analysis. Reconstitution solution consisted of 120 µL methanol-water (1:1, *v*/*v*) for analysis using a C18 column and acetonitrile-water (4:1, *v*/*v*) for analysis using a Hydrophilic Interaction Chromatography (HILIC) column.

For lipidomics, a 200 µL lipid internal standard custom mix (Supplementary Table S5b) was added to samples. After shaking for 30 min, samples were filtered under vacuum into a 96-deep well plate (Agilent, 2 mL depth).

### 2.5. Untargeted Metabolomics and Lipidomics

Untargeted metabolomic runs were performed in both positive and negative MS modes using two different sets of columns (HILIC and C18), each with their own set of mobile phases and Ultra-Performance Liquid Chromatography (UPLC) methods. The same high-resolution mass spectrometry (HRMS) method was used for all runs on a ZenoTOF 7600 Mass Spectrometer (AB SCIEX, Victoria, Australia). Untargeted lipidomic runs were also performed in positive and negative modes with their own mobile phases, LC method and HRMS method. HRMS was performed using a TripleTOF 5600 Mass Spectrometer (AB SCIEX, Victoria, Australia). See Supplementary Methods for full UPLC and HRMS methods.

To ensure an identifiable 1:1 match of features between discovery and validation data, all cohorts were processed together in the PeakView v2.2 software (SCIEX, Marlborough, MA, USA). Additional data processing was performed using SCIEX MultiQuant v3.0 and SCIEX OS v3.1.1.43 (SCIEX, Marlborough, MA, USA).

The discovery cohort of 2089 control and 104 case samples was profiled in 26 separate metabolomic and lipidomic runs, alongside an additional 89 carrier samples and 32 other samples with conditions flagged as NBS positives, but without genetic confirmation. Case, carrier and NBS positive-flagged samples were dispersed throughout each of the 26 runs alongside control samples of similar DBS card age, and injection order within each run was randomised. The validation cohort of 554 control and 76 case samples was profiled over 8 additional runs. The effects of running samples in separate batches was accounted for using internal standards and corresponding normalisation parameters during statistical analysis.

### 2.6. Statistical Analysis Methods

All statistical analyses were conducted in R v4.0 (R Foundation for Statistical Computing) [18]. For each dataset (metabolite type and ion mode), preprocessing was performed by removing features with >25% zero values, or with any zero-variance runs (all samples in a run had the same value), followed by log-transformation. Because substantial differences in distribution were observed between runs, log-transformed features were also standardised by normalising observations to the normal-sample mean and standard deviation of their run.

For analysis, case samples were categorised into six diagnostic subgroups (respiratory, multi-system, metabolic, endocrine, haematological, and other). For each diagnostic subgroup, elastic net regression models [19,20], with cross-validation, were used to select a subset of features for positive and negative mode of each metabolite type (i.e., 6 models for each subgroup). An alpha parameter of 0.5 (midway between lasso and ridge penalties) was chosen as a value which resulted in a manageable number of features without excessively strict selection that might increase the degree of overfitting. The feature subsets were the features selected at the minimum lambda (minimum cross-validation error).

Following this, the selected features were combined across diagnostic subgroups and positive and negative ion mode, resulting in three sets of features (lipids, C18, and HILIC). Using the means and covariances of these feature sets in the normal samples, we then calculated the Mahalanobis distance of all samples. This produced three multivariate distance metrics for each sample, which were used as predictors in a logistic regression model (for any type of disorder). The coefficients of the model, along with the normal-sample means and covariances, were retained for evaluation in the validation data.

To assess the performance of the predictive algorithm in the validation data, the three distance metrics were calculated for all validation samples using the retained normal-sample means and covariances from the discovery data, then the coefficients from the logistic regression model were used to calculate the log-odds (and therefore the predictive probability) of abnormality. The performance of the algorithm was then assessed in terms of discrimination (area under the ROC (receiver operator characteristic) curve), and sensitivity/specificity corresponding to various diagnostic thresholds defined in terms of percentiles of predicted probabilities, or actual predicted probabilities.

## 3. Results

For the development of the metabolomics screening algorithm, we established a discovery cohort comprised of 2089 control samples from presumably healthy newborns and 104 case samples with a confirmed genetic diagnosis, and a validation cohort of 559 healthy control samples and 76 case samples (Table 1, Supplementary Tables S2 and S3). Importantly, the composition of the validation and discovery cohort in terms of disease genes and groupings was quite different (Figure 2). Of the 59 unique genes represented among positive cases in the discovery cohort, only seven genes overlap with the 61 unique genes represented in the validation cohort. In addition, 88 samples with carrier status for an autosomal recessive condition and 32 samples that had been flagged by stNBS but with no molecular diagnosis, were included in these comparisons, including 20 cases of congenital hypothyroidism and 12 presumably false-positive NBS calls with an elevated metabolic biomarker but no molecular diagnosis (Supplementary Table S4). These formed a cohort of edge-cases for testing the performance of the novel metabolomic screening algorithm. In both the discovery and the validation cohort, the case samples were observed to have a lower mean birth weight and gestational age and were more likely to be bottle fed compared to the presumably healthy control samples (Table 1).

### 3.1. Known Biomarkers Showed Expected Elevation in Respective Case Samples

Comparison against internal standards confirmed elevation of metabolite signal in most expected samples, as well as in some cases for conditions not typically associated with these metabolites in NBS programmes (Table 2, Figure 3, Supplementary Figures S2–S4, Supplementary Tables S6–S8). Of the 22 cases with well characterised biomarkers for PKU, MCAD deficiency, VLCAD deficiency, carnitine-acylcarnitine translocase (CACT) deficiency, and propionic aciduria, 21 cases showed expected elevated metabolite signals, providing assurance that untargeted metabolomics could for most cases accurately identify at-risk cases (Table 2, Figure 3). One case of MCAD did only present with the expected elevated analyte/internal standard levels for carnitines at the 95th percentile, but not at the 99.5th percentile (Supplementary Table S6). In stNBS, this particular case showed the expected metabolite elevation only in the 9-day follow-up card, but not in the day 2 card. Some samples with diagnoses that have no known biomarkers in NBS programmes were also identified as having elevated signal for multiple different metabolites. Most notably, a case sample from the discovery cohort with a diagnosis of DiGeorge syndrome was elevated for three amino acids and four carnitines (Supplementary Table S6).

Among lipids and other non-standard biomarkers, four analytes stood out which identified cases of Zellweger syndrome, propionic aciduria, trisomy 21, haemophilia A, mitochondrial respiratory chain disorders, and a sickle cell disease carrier (Supplementary Table S8).

### 3.2. Feature Selection in Untargeted Metabolomics and Lipidomics

Statistical analyses were performed to select those features that contain most of the variation between control and case samples (Table 3). A total of 1115 features were selected from an initial 30,565 features and included in the elastic net models. The number of features in the initial data, those removed, and those included for modelling is available in Supplementary Table S9.

Performance of the model and selected features was evaluated in the discovery, validation and edge case data (Figure 4). In the discovery data, the logistic regression model had excellent discrimination (Area Under the ROC curve 0.994, 95% CI: 0.989, 1.000) (Figure 4(1c)). At the 95th percentile of predicted probability (classifying the highest 5% of values as cases), sensitivity was 90.57% with a specificity of 99.55% (Figure 4(1b), Supplementary Table S10). Individual predicted probability values of being an ‘outlier’ or ‘case’ are provided for each sample in Supplementary Table S11. As the performance is being evaluated in the same dataset used to develop the model, predictive performance is likely overestimated.

In the validation samples and using the predictive model developed using the discovery samples only, case samples tended to be outliers (sometimes extreme outliers) in the distribution of predicted probability (Figure 4(2a)). As expected, predictive performance in the validation data was substantially decreased in comparison to the discovery data, but discrimination was still good, with an Area Under the ROC curve of 0.799 (95% CI: 0.736, 0.863) (Figure 4(2c)). Using the 95th percentile of predicted probability as the diagnostic cutoff would result in a sensitivity of 36.84% and a specificity of 99.28%, while using an actual 95% predicted probability as the cutoff would result in a sensitivity of 40.79% and a specificity of 99.10%. (Figure 4(2b), Supplementary Table S10; individual predicted probability values of being ‘a case’ are provided for each sample in Supplementary Table S12).

The predictive model developed here also flagged many carriers and other screen-positive NBS samples such as carriers of CF or alpha thalassaemia and cases of congenital hypothyroidism (Figure 4(3a), Supplementary Tables S10 and S13).

Additional exploratory analyses were carried out to assess the impact of using the discovery data means and covariances for calculation of Mahalanobis distances in the validation data, rather than calculating these parameters from the validation data itself (Supplementary Tables S11 and S12). The reasons for using discovery data parameters were firstly, that the sample size was far larger, and secondly, that in prospective cohorts, the status of the sample (diagnosed case vs. control) will not be known so that it will not be possible to calculate means and covariances for control samples only. However, if the distributions of the selected features differed substantially between discovery and validation data, use of the discovery data distribution could affect predictive performance.

We found that in the validation data, diagnosed cases were more extreme outliers in relation to the multivariate distributions of the control samples, than in the original discovery cohort. Calculating log odds using these distance metrics in the logistic regression equation (from the discovery data) resulted in perfect discrimination (Area Under the ROC Curve of 1) and sensitivity of 98.68%, with perfect specificity, when using a predicted probability of 97.50% as a diagnostic cutoff. (When using both control and case samples to calculate the multivariate distributions, AUROC was 0.773, and all predicted probabilities were <5% (Supplementary Table S10, Supplementary Figures S5 and S6).

## 4. Discussion

In this study, we aimed to establish a metabolomic reference profile which could be used to screen DBS samples to detect outlier profiles that may be suggestive of an underlying genetic condition. To achieve this, we performed untargeted metabolomic and lipidomic analyses on almost 3000 retrospective DBS samples. Significant features differentiating control from case samples were selected using multi-variate statistical methods. These features and the calculated model were validated using an independent, retrospective validation cohort to assess the model performance.

### 4.1. Review of Known and Emerging Biomarkers

To determine the efficacy of the untargeted metabolomic methods to accurately quantify known biomarkers, we reviewed area ratio signals for well-established biomarkers in conditions routinely screened for in NBS. Using deuterated forms of biomarkers used in NBS as internal standards, untargeted metabolomics was able to correctly identify 21 of 22 cases with area ratios greater than the 99.5th percentile of the discovery cohort control samples. Further analysis determined that the one case not flagged by the algorithm was also not identified through traditional NBS until later in life, suggesting that the untargeted method performed at a similar level to traditional NBS.

Further review of the discovery and validation cohorts using emerging biomarkers from the literature did not provide clear discrimination between control and case samples using untargeted metabolomic data. Many biomarkers drawn from the literature did not clearly show a link between the disease for which they were supposedly a marker, and many biomarkers had poor peak signal. This suggests that either singular biomarkers alone were not sufficient to detect cases from controls, or that the analytical conditions were not suitable for these markers, further highlighting the importance of using more complex multi-variate models and a more comprehensive set of biomarkers for disease screening. Review of the lipidomic data for discovery and validation cohorts did, however, provide some discrimination. In the lipidomic analysis of the discovery cohort, Chol Ester(28:5) was highly elevated in a Zellweger syndrome case sample. This syndrome is a peroxisomal condition that has been associated with intracellular cholesterol transport, resulting in insufficient ciliary cholesterol levels [21]. This could also result in build-up of other cholesterol-related molecules such as Chol Ester(28:5). A similarly extreme area ratio for this sample was also found for LPC(C26:0). LPC(C26:0) is a known marker for peroxisomal conditions like Zellweger syndrome, including in DBS studies [22,23].

The other notable outliers found during lipidomic analysis were SM(35:1) for propionic aciduria and PC(O-38:5) for trisomy 21. The propionic aciduria case sample was also a metabolomic outlier as expected due to propionyl-CoA carboxylase deficiency resulting in a build-up of propionyl carnitine [24]. The concentration of SM(35:1), a sphingomyelin phospholipid, could also be affected by the deficiency in PCC, giving rise to an elevated area ratio compared to other samples. For the analyte PC(O-38:5), more specifically determined to be PC(P-18:0/20:4), one of two trisomy 21 case samples in the discovery cohort was a clear outlier. Changes in lipid profiles have been previously noted in patients with trisomy 21 [25].

### 4.2. Predictive Performance of the Multi-Variate Model in Retrospective Samples

The performance of the predictive model in the discovery and validation cohorts was high, including for discriminating cases which may not be expected to have a metabolomic signature, such as non-metabolic conditions. On the other hand, some case samples representing the very same condition, showed highly different predictive probabilities, suggesting that in its current form, the algorithm may miss identifying some cases for reasons that are currently not understood, but which may be related to sample quality or other factors contributing to the metabolomic/lipidomic state of the newborn sample in question.

Investigation into the different genetic condition subgroups did not reveal any clear patterns to suggest which conditions or condition subgroups had higher discrimination in this model, compared to others. All metabolic and multi-system conditions showed high predictive probabilities, but so did most other conditions assessed here. On the other hand, some conditions represented by multiple case samples showed different discrimination performance across samples. For example, in the discovery cohort, two samples with a prior diagnosis of thyroid dyshormonogenesis 6 (*DUOX2* gene) showed a very high predictive probability of >0.993 in the model, while one other sample with the same condition showed a low predictive probability of 0.159 in the same model. Similarly, six cases with a prior diagnosis of haemophilia A (*F8* gene) had high predictive probabilities (ranging from 0.793 to 0.999), while one other sample with the same condition showed a low predictive probability of 0.109 in the same model. Finally, of the 16 samples with a prior diagnosis of cystic fibrosis (*CFTR* gene), 15 showed high predictive probabilities but one case sample was not identified, with a predictive probability of 0.005.

Similarly, sample performance in the validation cohort differed among samples without any clear pattern that could be attributed to individual conditions or condition subgroups and sometimes, among cases of the same condition. For example, of three cases with a prior genetic diagnosis of sickle cell disease (*HBB* gene), only one sample showed high predictive probability in the model. Interestingly, and despite the vastly different gene and condition composition of the validation cohort compared to the discovery cohort, many conditions represented only in the validation cohort still had high predictive probabilities and this included many non-metabolic conditions, such as Rubinstein-Taybi syndrome 2 (*EP300* gene), achondroplasia syndrome (*FGFR3* gene), spinal muscular atrophy (*SMA* gene), long QT syndrome (*KCNQ1* gene), and Noonan syndrome (*LZTR1* gene), among others. Overall, and at a 95% predicted probability threshold, the model’s sensitivity in the validation cohort was 40.79% with a specificity of 99.10%. Finally, we purposefully included some carriers and other stNBS screen-positive cases to assess the performance of the model beyond the target conditions of interest. As expected, the model also flagged many of these samples as outliers. Of the 89 carrier cases, 26 cases had a predicted probability >95% and of the 23 other cases flagged by stNBS, 12 cases had a predicted probability >95%.

The algorithm constructed here using a multi-step process and validated in an independent cohort of control and case samples (validation cohort), shows significant potential for development into a screening tool for a range of genetic conditions in newborns. While predictive performance in the validation dataset was not at the level required for adoption as a routine screening tool, there is reason to believe that further improvements can be made.

In particular, we used the normal-sample means and covariances from the discovery dataset to define the reference distribution against which the validation samples were measured; however further analyses showed that the cases in the validation data were more extreme outliers in relation to the normal-sample means and covariances. This suggests that some of the decreased predictive performance may be due to differences in the normal-sample distribution between the discovery and validation datasets, and that predictive performance could be improved by constructing the normal profile from contemporaneous cohorts.

In a prospective population, scientists will be blind to the case/control status of prospective DBS samples, and will therefore not be able to ensure that the reference profile is constructed only from control samples. However, the expected proportion of cases in the population is extremely small, whereas cases were deliberately oversampled in the discovery and validation cohorts. Therefore, we expect that the whole-cohort means and covariances may be close approximations to the normal-sample distributions.

### 4.3. Limitations

Several limitations must be noted in the present study. Potential confounding effects of sample age and of gestational age of the infant could not be fully excluded here. As noted above, the age of the DBS cards affected the behaviour of metabolites in ways that could not be fully accounted for using statistical methods. While efforts were thus made to minimise these effects by restricting the time range of collection of samples (2019 to 2024 only), and by standardising features to run-specific means and standard deviations, there is a possibility of residual confounding effects impacting the algorithm, and hence the predictive performance in future prospective samples. Similarly, because of the way in which the healthy controls or reference samples were selected (specifically, the criterion of 39–42 weeks gestational age at delivery), preterm birth may be a strong confounder for case status prediction. It is well understood that screening results in stNBS can be affected by gestational age. While it is possible that gestational age similarly affects the behaviour of metabolites in the current model, we were unable to account for this in the present study. Similarly to standard NBS, additional DBS cards collected at different time points may be required, and results may need to be interpreted in conjunction with clinical findings and other laboratory investigations in this context. Some conditions may have been inadvertently included in our reference set due to these cases not having been diagnosed by orthogonal or clinical pathways at the time of this study. We would expect this to only affect a small proportion of babies in this cohort and to not significantly skew the reference set distribution used in the model.

In addition, ethnic background and carrier status are known to affect current stNBS and can result in false-positive screening calls [26,27,28]. It is likely that the model developed here will suffer from similar confounders and, indeed, many carriers, purposefully included here to assess this, showed a high predictive probability of disease in our model. Importantly, however, just as in stNBS, confirmatory testing can be performed to either confirm or dismiss a screen-positive result. At present, at the 95% threshold, the specificity of the model was set high to avoid false-positive calls, which in a prospective setting would result in family recall, at the expense of sensitivity. This may be an acceptable screening approach while the specificity/sensitivity trade-off of this model can be further optimised.

### 4.4. Future Directions

The novel screen presented here appears to hold promise as a complementary test alongside current NBS methods, for example as a triaging tool to identify newborns at higher risk of genetic conditions who could then be offered gNBS in a multi-omic approach. For the development of this screen and despite best efforts to include as many case samples as possible, many conditions of interest for inclusion in gNBS programmes could not be represented in the present study due to their rarity and a lack of locally available DBS samples. For this reason, and rather than attempt to develop biomarkers for a vast range of diverse genetic conditions, we took the approach to characterise a ‘reference profile’ of presumably healthy newborns, so that the profiles of a diverse range of genetic conditions could be compared against this reference profile. While not yet tested systematically, the present results are promising, and the predictive model developed here appears to extrapolate to many conditions not represented in the discovery cohort in which the model was developed. It is hoped that this will hold true for many other conditions of interest for gNBS inclusion which could not be assessed here. Larger cohort studies will be needed to test this hypothesis.

The feasibility of performing *untargeted* metabolomic and lipidomic profiling in a large number of samples was found to be a limiting factor if this was to be implemented as a routine test due to the high instrument and run time requirements. To facilitate larger-scale testing, including population-scale screening, we thus decided to explore the use of a semi-targeted metabolomic/lipidomic screening assay in prospective cases, after the initial untargeted discovery study presented here. To this end, the >1100 features selected here to generate the reference profile need to be optimised for inclusion in a semi-targeted screening assay. We expect that some features will actually represent isotopes of the same metabolite or the same metabolite corresponding to different features in the lipid, C18, and HILIC datasets. Other selected features may not correspond to true metabolites at all, or may not be suitable for inclusion in semi-targeted metabolomics, all the above likely resulting in a further reduction of the final feature set for inclusion in future semi-targeted assays.

As part of the ongoing NewbornsInSA study, we are now optimising the features selected in this model for use in a semi-targeted assay on a triple-quadrupole mass spectrometer. If successful, this approach will be more amenable to screening large numbers of samples, including at population scale, compared to the untargeted metabolomic and lipidomic methods performed here to develop the model. The intent is to integrate the metabolomic/lipidomic screening with genomic newborn screening as part of the broader prospective NewbornsInSA study in which South Australian families are invited to participate [17]. As part of this work, we will examine the performance of the metabolomics screen developed here, alongside gNBS, in a prospectively recruited newborn cohort, including determination of false-positive and false-negative rates, laboratory workflow, and cost implications, all of which would be essential considerations for clinical implementation.

Beyond our study, the data and approach presented here have identified many opportunities for future research. For example, future work may review the performance of metabolomic and lipidomic features to distinguish different condition subgroups, in particular metabolic vs. non-metabolic conditions to determine generalisability of the model. So far, it appears that the algorithm can successfully discriminate case samples across a very broad range of condition subgroups, and this would be interesting to examine in a larger and even more diverse cohort, including whether the model performance differs for different metabolite subtypes (HILIC, C18, lipids) for different disease subgroups. For case samples that could not be identified, it would be interesting to assess if there was a difference in the age of onset of the disease, as possibly the newborn DBS sample may not be suitable to identify conditions with a disease onset later in childhood. One concern that has been raised with genomic newborn screening studies is that the presence of genetic variants may lead to over-estimation of disease prevalence and possibly over-treatment of pre-symptomatic cases, as disease onset may likely depend on many other, including non-genetic, factors, such as genetic background, environment, and lifestyle. The integration of metabolomics and lipidomics with genomic data may prove helpful in this context to obtain a more holistic view of disease status. Finally, metabolomic and lipidomic features may be used as a monitoring tool in response to treatment or for the identification of potential new therapies. Novel algorithms, including the use of artificial intelligence approaches, may be needed to assist in the identification of therapy opportunities from metabolomic and lipidomic data.

## 5. Conclusions

Early detection and prevention of health conditions are key aims of modern healthcare. For rare disease research, however, the challenge lies in the diversity and rarity of the individual genetic conditions which make the development of effective screening approaches difficult. Multi-omic approaches offer new opportunities to address these healthcare needs and may allow screening for a broad range of rare genetic conditions, without requiring the development of individual biomarker-based methods.

## Figures and Tables

**Figure 1:**
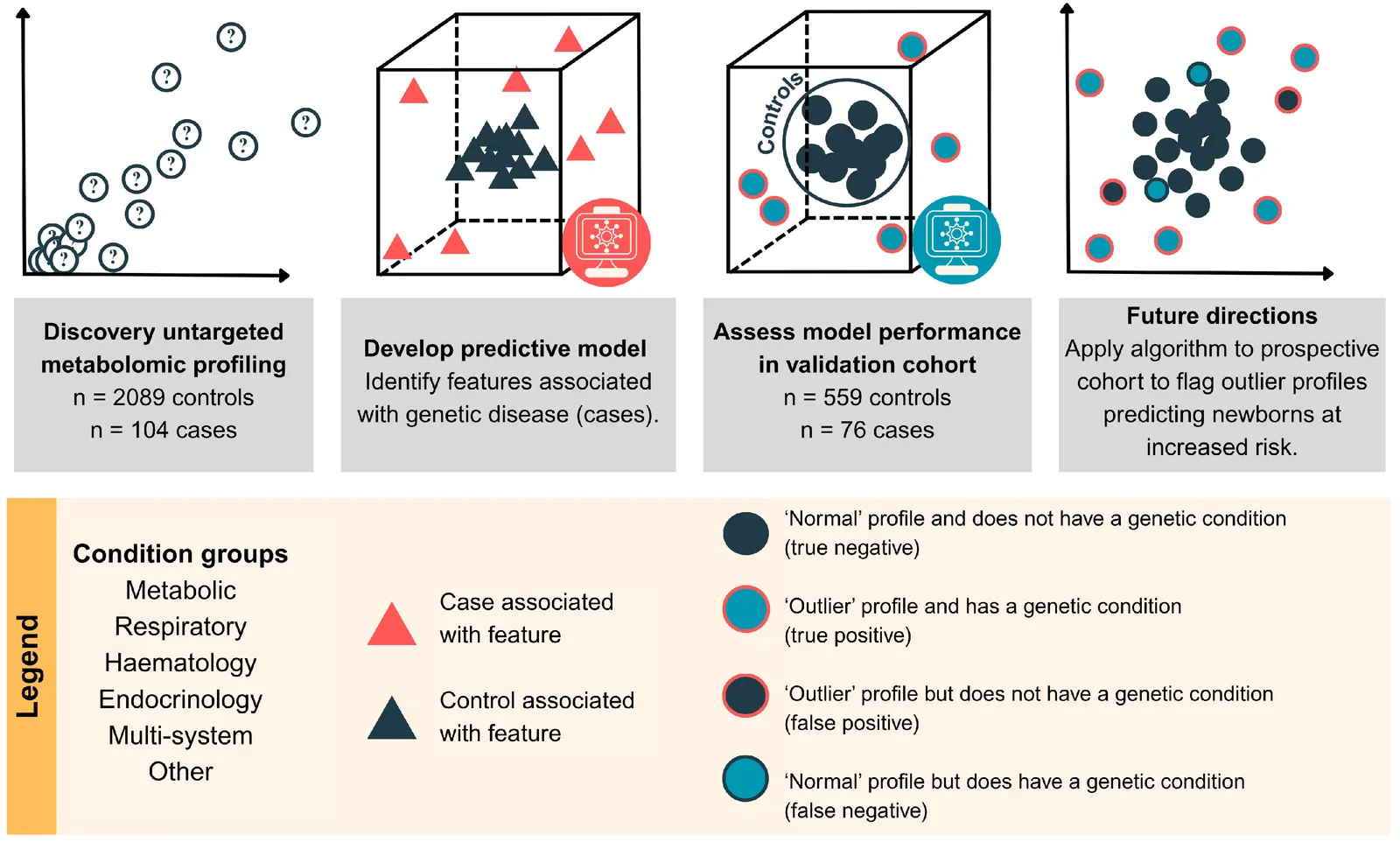
Development of a novel metabolomic screening method using untargeted metabolomic profiling of dried bloodspots. Untargeted metabolomic profiling of a discovery cohort comprising cases with genetic conditions and controls was used to identify key metabolomic features which formulate a ‘reference profile’. A statistical algorithm based on the relative variance of these key features was designed and applied to a validation cohort to assess the algorithm’s performance. The algorithm is intended for future application in a prospective research study of newborn bloodspot screening to identify newborns at increased risk of genetic conditions.

**Figure 2:**
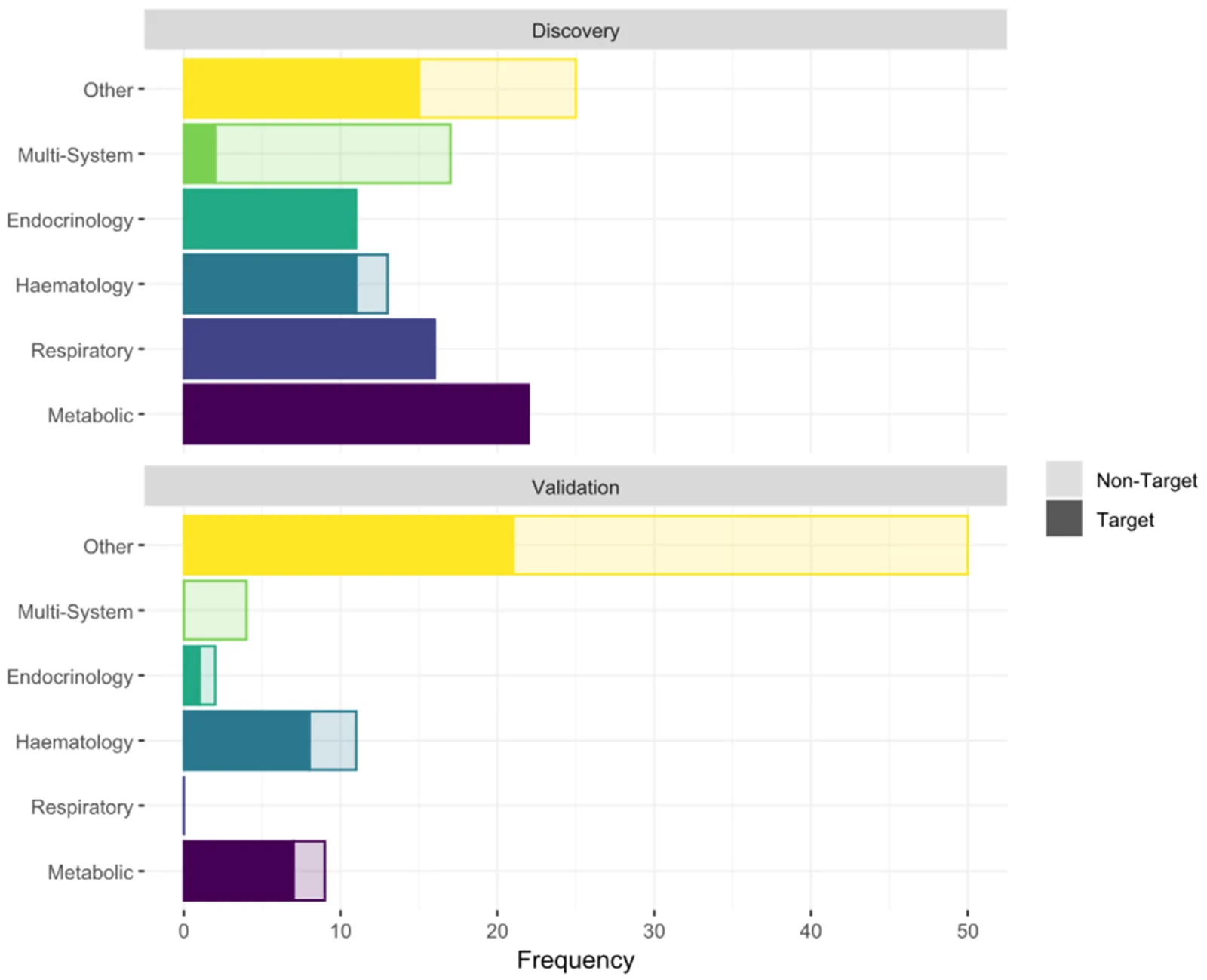
Disease group composition among positive cases in the discovery and validation cohorts. Proportion of target genes versus non-target genes across disease groups included in the discovery cohort and validation cohort. Target conditions refer to those prioritised in the NewbornsInSA study based on early onset in childhood and with a recommended treatment, while non-target conditions refer to other genetic conditions, not currently being assessed for genomic or multi-omic newborn screening.

**Figure 3:**
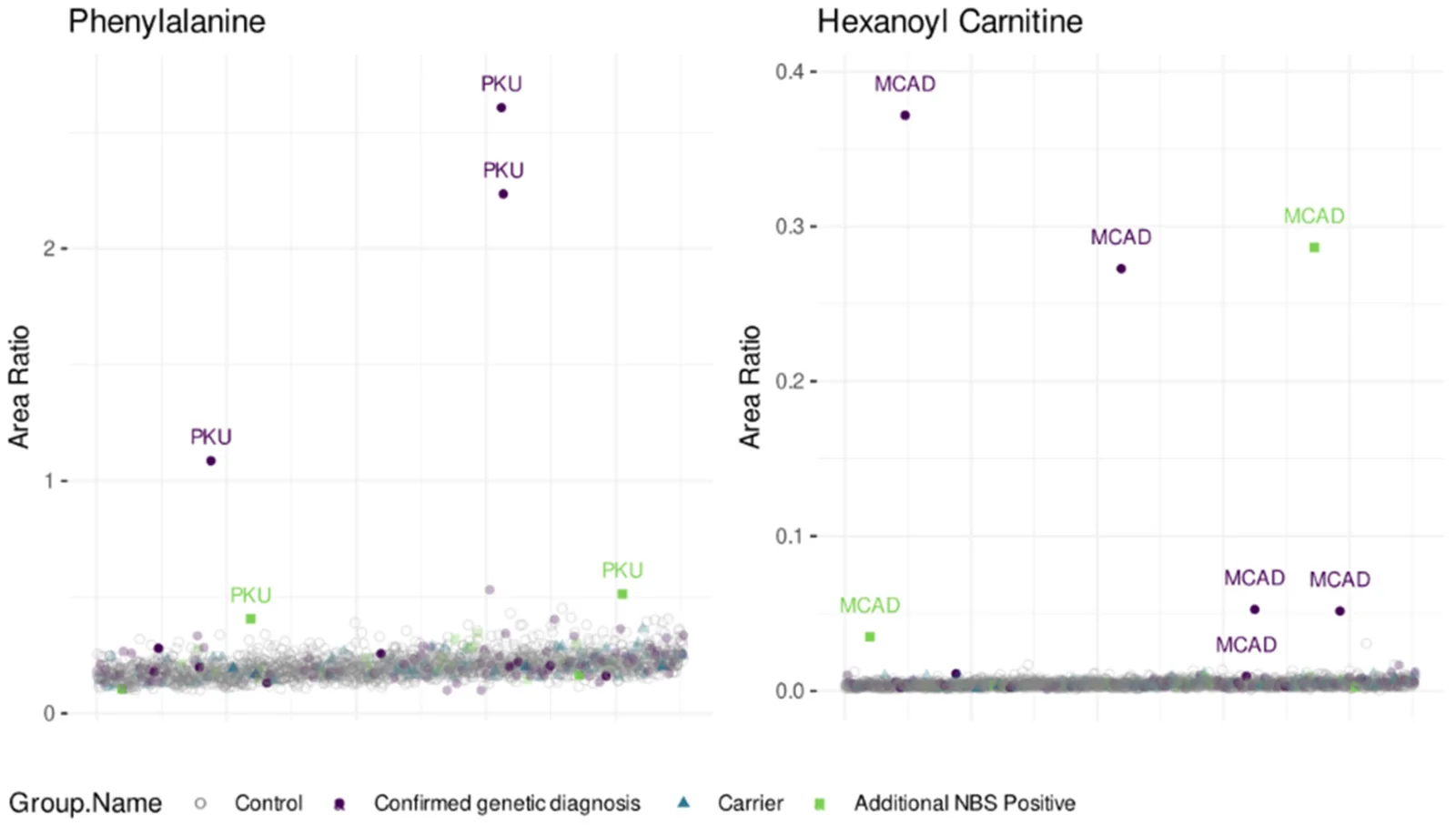
Area ratio between analyte/internal standard measured by untargeted metabolomics for all samples within the discovery cohort, including genetic cases, carriers and additional NBS positives. Samples with expected elevated biomarkers, based on known prior diagnosis with the condition, are highlighted. Also highlighted are additional samples that were flagged as positive through the newborn screening programme. Metabolomics analysis was performed in 2313 retrospective samples using a QTOF-MS in positive mode and a C18 column.

**Figure 4:**
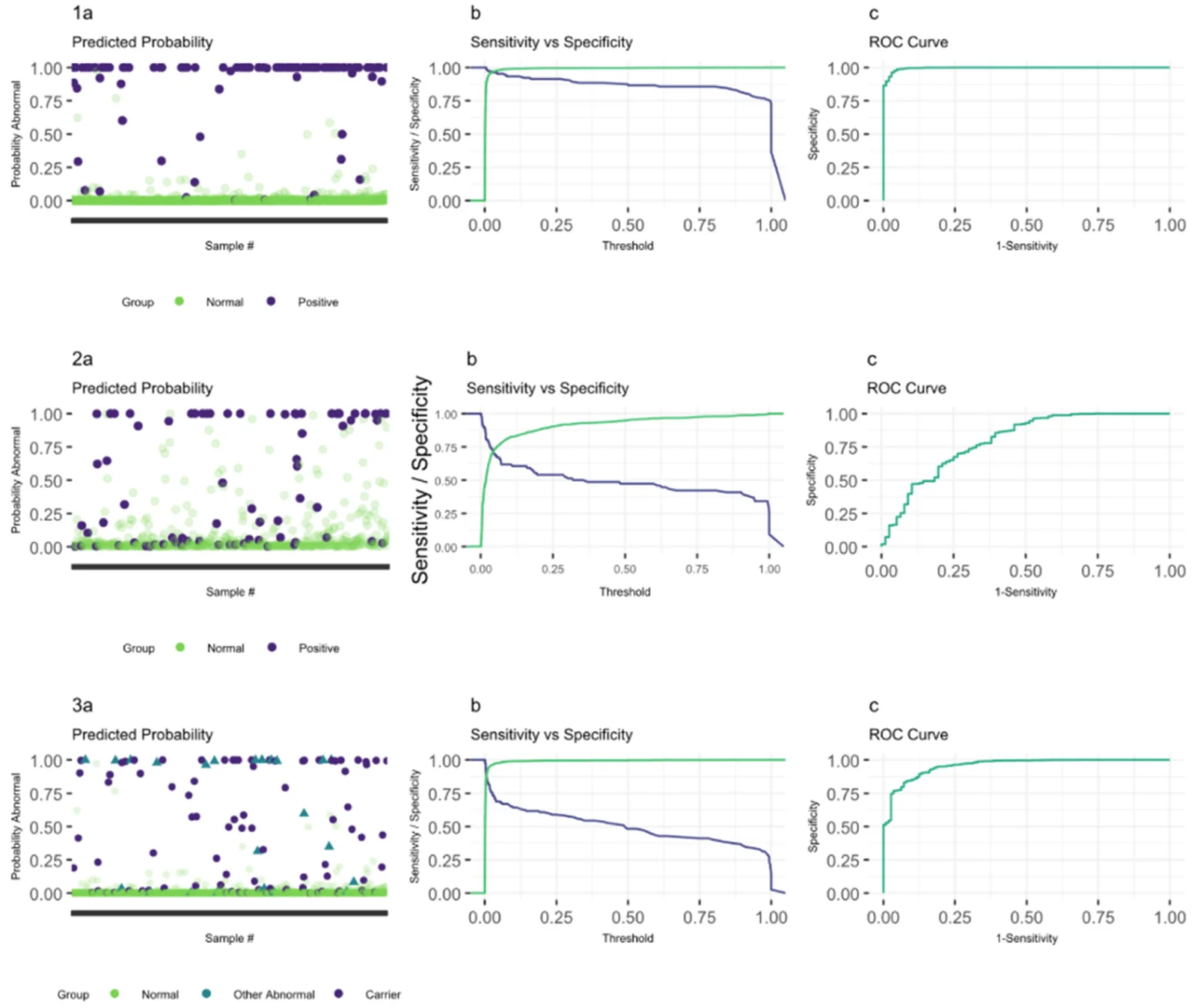
Plots of predicted probability distributions, sensitivity and specificity across the range of diagnostic thresholds and ROC curve, calculated using the model and algorithm developed using the discovery cohort data. (**1a**) Predicted probability for discovery data. (**1b**) Sensitivity (purple line) and specificity (green line) across the range of diagnostic thresholds for discovery data. (**1c**) ROC curve for discovery data. (**2a**) Predicted probability for validation data. (**2b**) Sensitivity and specificity across the range of diagnostic thresholds for validation data. (**2c**) ROC curve for validation data. (**3a**) Predicted probability for edge case data (carriers and additional NBS positives). (**3b**) Sensitivity and specificity across the range of diagnostic thresholds for edge case data. (**3c**) ROC curve for edge case data.

**Table 1:** Cohort demographics for healthy controls and cases with genetic conditions, as well as genetic carriers and additional NBS positives. Additional NBS Positives refer to samples with elevated metabolites recorded in standard newborn screening but no molecular diagnosis.

	Discovery Cohort	Validation Cohort	Carriers	Additional NBS Positives
**Healthy controls**	**2089**	**559**		
DBS collection dates	2019–2023	2019–2024		
Sex				
Males	49.35%	50.45%		
Females	50.65%	49.55%		
Gestational Age (weeks)—Mean (std)	39.53 (0.68)	39.29 (0.87)		
Birth weight (kg)—Mean (std)	3.43 (0.32)	3.41 (0.32)		
Feeding type				
Breast fed	94.06%	93.21%		
Bottle fed	5.46%	6.43%		
**Cases (Unique Genetic conditions)**	**104 (59)**	**76 (61)**	**88 (10)**	**32 (8)**
Metabolic	22 (17)	9 (8)	8 (5)	12 (7)
Respiratory	16 (1)	0 (0)	59 (1)	0 (0)
Haematology	13 (3)	11 (8)	19 (4)	0 (0)
Endocrinology	11 (9)	2 (2)	0 (0)	20 (1)
Multi-system	17 (10)	4 (3)	0 (0)	0 (0)
Other	25 (19)	50 (40)	0 (0)	0 (0)
DBS collection dates	2019–2023	2019–2024	2019–2023	2019–2022
Sex				
Males	48.08%	48.00%	45.45%	37.50%
Females	51.92%	52.00%	54.55%	62.50%
Gestational Age (weeks)—Mean (std)	37.87 (2.17)	37.35 (3.36)	38.42 (1.53)	38.37 (2.76)
Birth weight (kg)—Mean (std)	3.15 (0.70)	3.13 (0.72)	3.29 (0.59)	3.19 (0.61)
Feeding type				
Breast fed	80.8%	70.67%	92.05%	78.13%
Bottle fed	8.65%	17.33%	5.68%	15.63%
**Total number DBS samples**	**2948**			

**Table 2:** Percentage of known positive cases identified by expected elevated biomarkers in untargeted metabolomics when compared against internal standards. Elevated signal was defined as an area ratio of analyte/internal standard above the ≥99.5th percentile. Biomarker levels were measured among >30,000 features using untargeted metabolomics.

Condition [Biomarker]	Discovery Cohort (% True Positive Cases Identified)	Validation Cohort (% True Positive Cases Identified)	Carriers (% Cases Identified)	Additional NBS Positive (% Cases Identified)
PKU [phenylalanine]	100% (3 of 3)	100% (1 of 1)	n/a	100% (2 of 2)
MCAD [C6, C8 and C10 carnitines]	80% (4 of 5)	n/a	n/a	100% (3 of 3)
VLCAD [C10, C12 and C14 carnitines]	100% (1 of 1)	n/a	100% (3 of 3)	100% (5 of 5)
CACT [C12, C14, C16 and C18 carnitines]	100% (1 of 1)	n/a	n/a	n/a
Propionic aciduria [C3 carnitine]	100% (1 of 1)	n/a	n/a	n/a

n/a = not applicable.

**Table 3:** Number of features selected for each metabolite type, ion mode and diagnostic subgroup.

Group	Lipid +	Lipid −	C18 +	C18 −	HILIC +	HILIC −	Total
Respiratory	33	17	34	43	37	30	194
Metabolic	80	97	58	46	65	89	435
Haematologic	5	2	14	5	5	33	64
Multi-System	44	49	40	37	45	92	307
Endocrine	1	1	1	31	1	3	38
Other	1	28	3	7	37	1	77
Total	164	194	150	169	190	248	1115

## Data Availability

Data are contained within the article or supplementary material. Variant-level genomic data available on request due to privacy restrictions.

## Supplementary Materials

The following supporting information can be downloaded at: https://www.mdpi.com/article/10.3390/ijns12040077/s1, Supplementary Table S1: NewbornsInSA core and secondary gene list V1.2. Supplementary Table S2: Case details (discovery cohort). Supplementary Table S3: Case details (validation cohort). Supplementary Table S4: Case details (Carriers and Additional NBS Positives cohort). Supplementary Table S5a: Internal standards for metabolomics. Supplementary Table S5b: Internal standards for lipidomics. Supplementary Table S6: Comparison against internal standards—metabolomics (discovery cohort). Supplementary Table S7: Comparison against internal standards—metabolomics (validation cohort). Supplementary Table S8: Comparison against internal standards—lipidomics (discovery cohort). Supplementary Table S9: Number of features in the initial metabolomics/lipidomics data, those removed, and those included for modelling. Supplementary Table S10: Sensitivity and specificity corresponding to different diagnostic thresholds in the discovery, validation and edge case data. Supplementary Table S11: Predicted probability of the logistic regression model in the discovery cohort in which the model was developed. Supplementary Table S12: Predicted probability of the logistic regression model in the validation cohort. Supplementary Table S13: Predicted probability of the logistic regression model in Carriers and Additional stNBS Positive samples. Supplementary Figure S1: Stability experiment on DBS. Supplementary Figure S2: Area ratio between internal standard and analyte values measured with QTOF-MS in positive mode using a C18 column for all samples used in retrospective study. Supplementary Figure S3: Area ratio between internal standard and analyte values measured with QTOF-MS in positive mode using a HILIC column for all samples used in retrospective study. Supplementary Figure S4: Area ratio between internal standard and analyte values measured with QTOF-MS in negative mode using a HILIC column for all samples used in retrospective study. Supplementary Figure S5: Mahalanobis distances calculated for the validation data. Supplementary Figure S6: Area under the ROC curve for predicted probability in the validation data. Supplementary Methods—Untargeted metabolomics and lipidomics using HRMS. Supplementary Analysis—Comparison against internal standards.

## Abbreviations

The following abbreviations are used in this manuscript:

NBS	Newborn bloodspot screening
PKU	Phenylketonuria
IEM	Inborn errors of metabolism
CF	Cystic fibrosis
SMA	Spinal muscular atrophy
SCID	Severe combined immunodeficiency
DBS	Dried blood spot
NGS	Next-generation sequencing
stNBS	Standard-of-care NBS
WGS	Whole-genome sequencing
MCAD	Medium-chain acyl-CoA dehydrogenase
VLCAD	Very long-chain acyl-CoA dehydrogenase
LC-MS	Liquid chromatography-mass spectrometry
HILIC	Hydrophilic interaction chromatography
UPLC	Ultra-performance liquid chromatography
HRMS	High-resolution mass spectrometry
CACT	Carnitine-acylcarnitine translocase
ROC	Receiver operating characteristic
AUROC	Area under receiver operating characteristic
gNBS	Genomic NBS
