# Supplementary material for: Expanding Conditions for Newborn Screening—Findings from an Untargeted Metabolomics and Lipidomics Discovery Study
Source: Int J Neonatal Screen. 2026 Sep 24;12(4):77. doi: 10.3390/ijns12040077 (PMC13692527; doi:10.3390/ijns12040077)
Supplement: Supplementary file 1 [file IJNS-12-00077-s001.zip › Ashenden et al Supplementary Figures and Methods (1).pdf]

Supplementary Figures and Methods

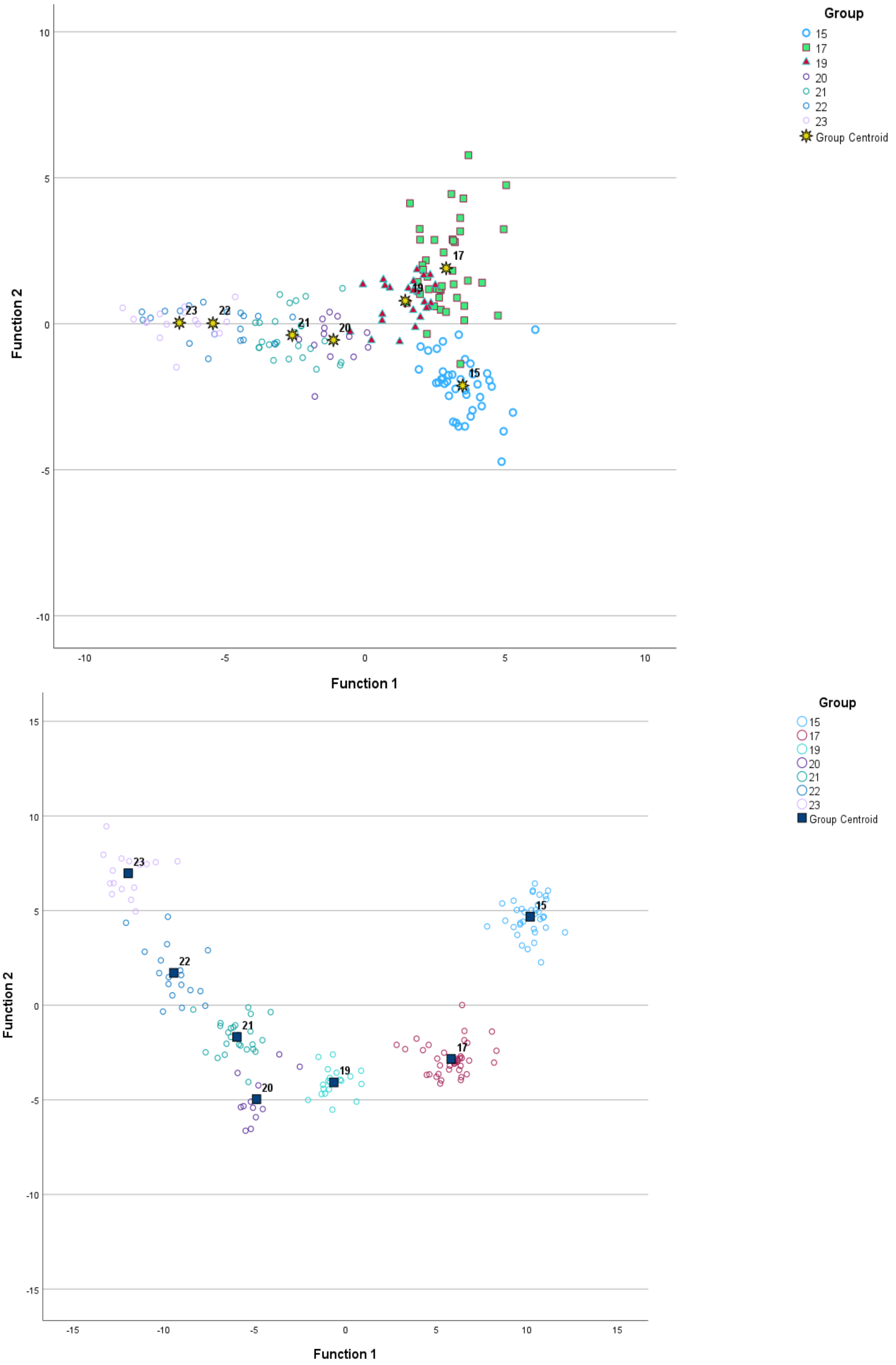

Supplementary Figure S1: Stability experiment on DBS. Principal component analysis of metabolomic data from C18 +ve (top) and HILIC -ve (bottom) QTOF-MS runs of samples from 2015-2023.

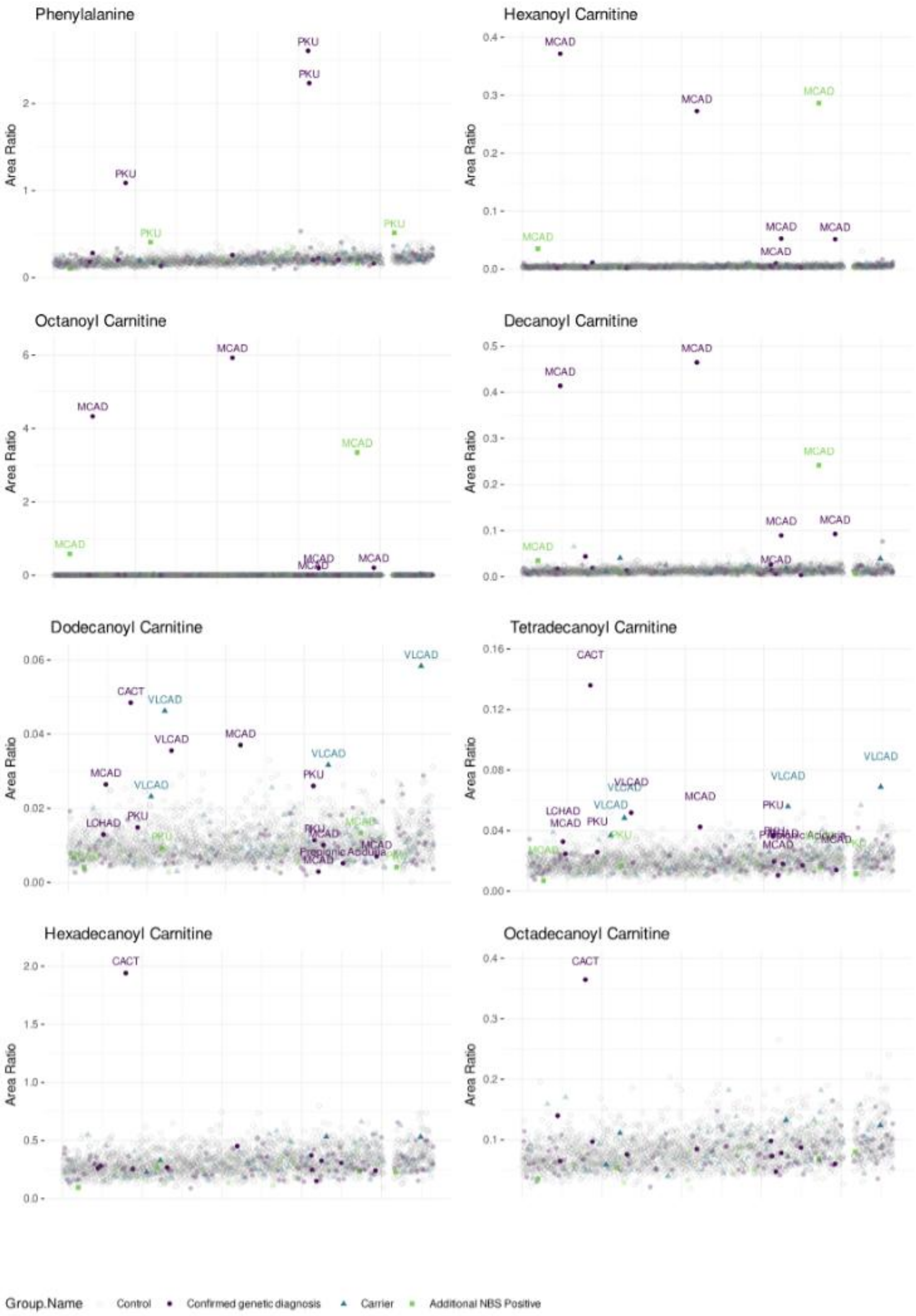

Supplementary Figure S2: Area ratio between internal standard and analyte values measured with QTOF-MS in positive mode using a C18 column for all samples used in retrospective study.

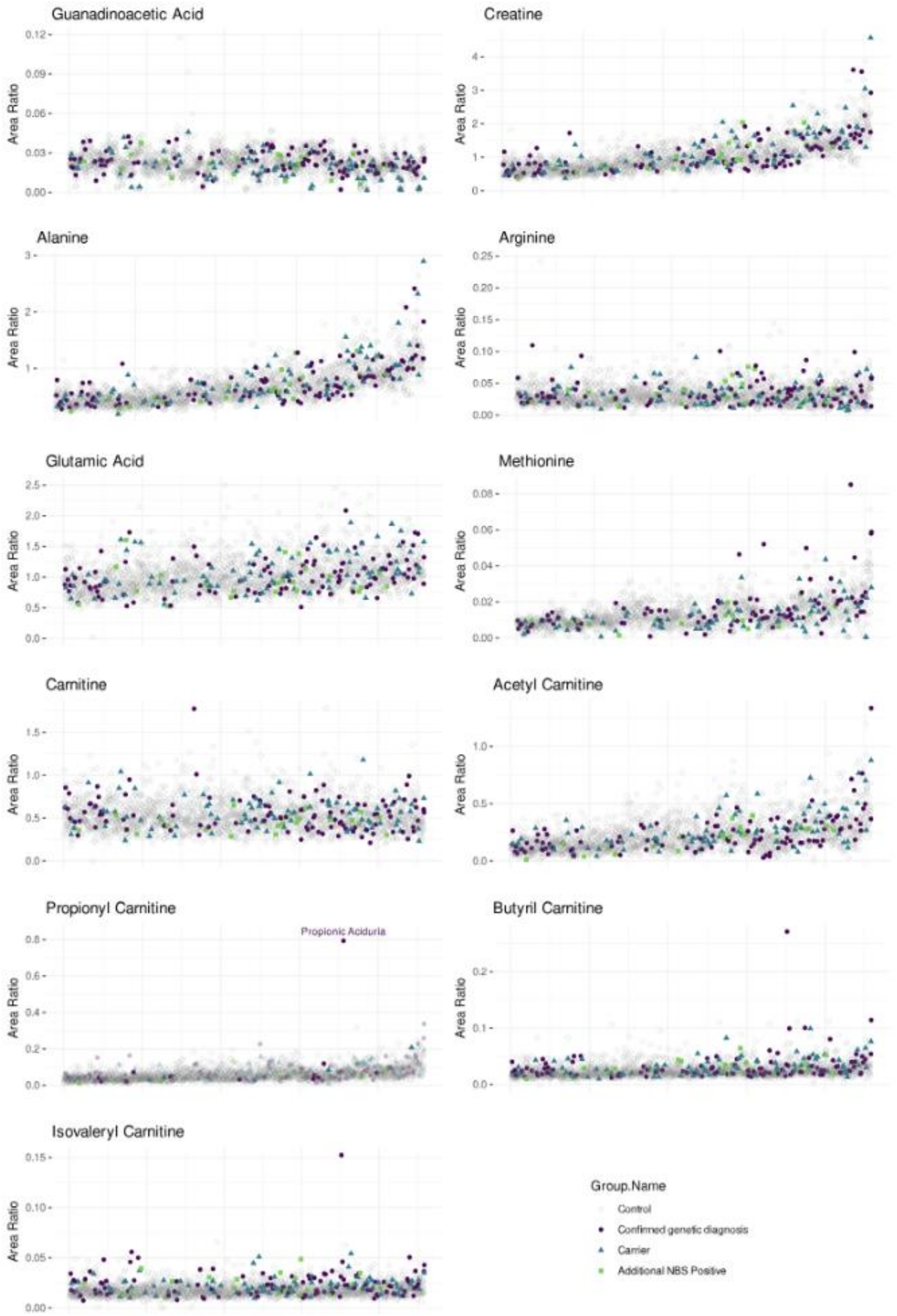

Supplementary Figure S3: Area ratio between internal standard and analyte values measured with QTOF-MS in positive mode using a HILIC column for all samples used in retrospective study.

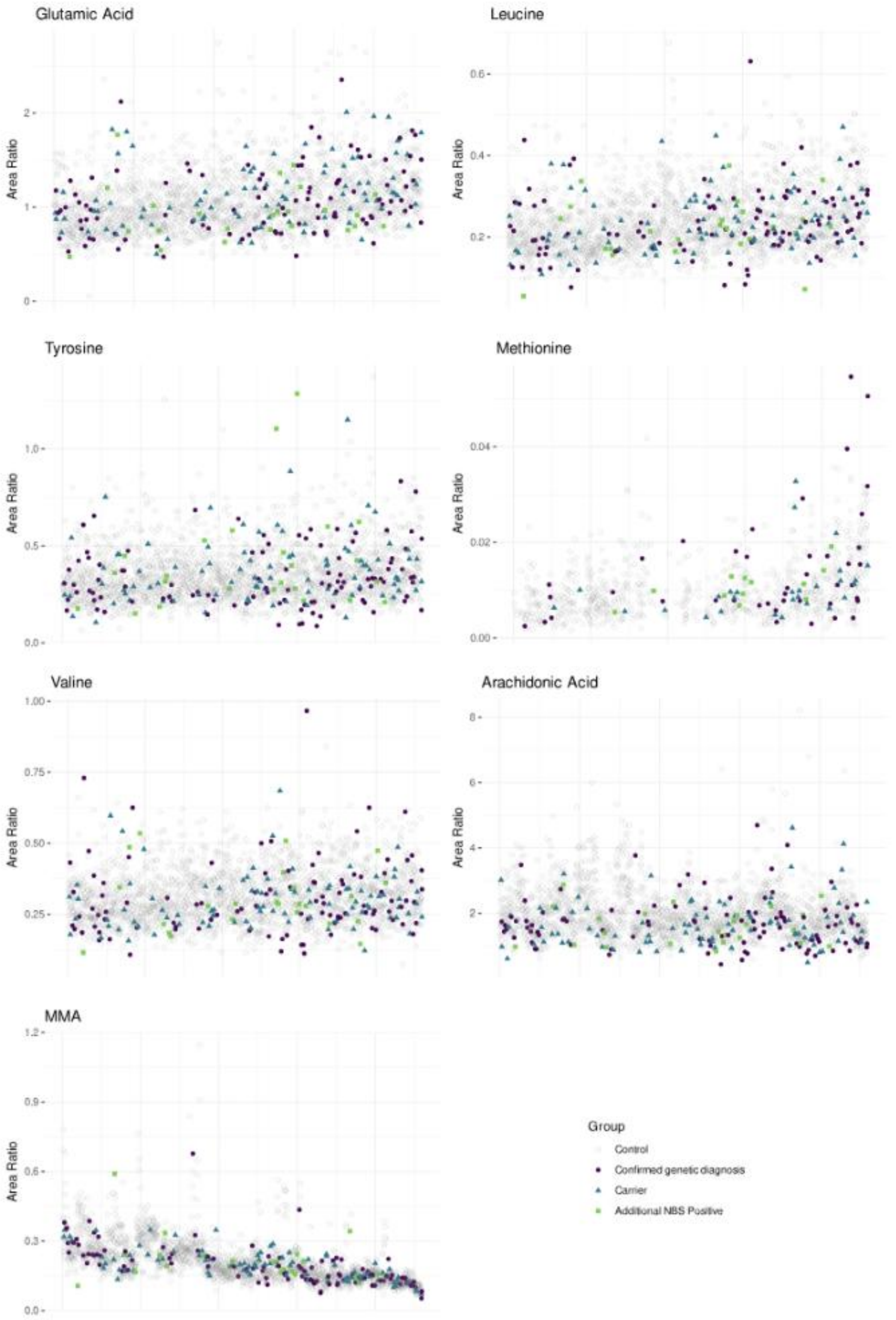

Supplementary Figure S4: Area ratio between internal standard and analyte values measured with QTOF-MS in negative mode using a HILIC column for all samples used in retrospective study.

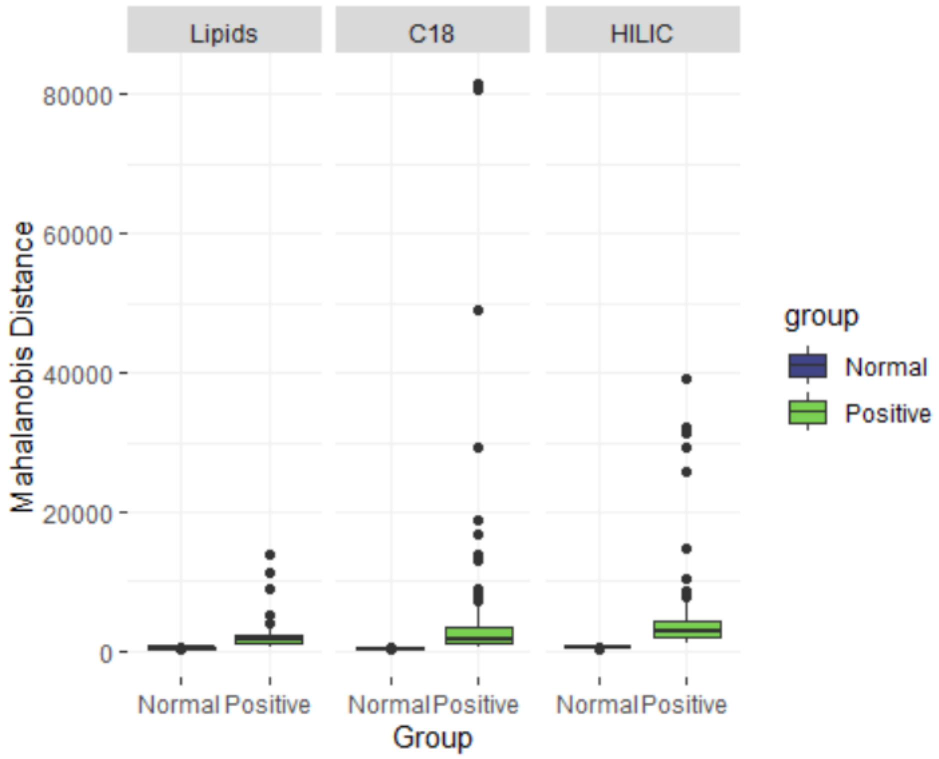

Supplementary Figure S5: Mahalanobis distances calculated for the validation data, relative to the distribution of normal samples in the validation data.

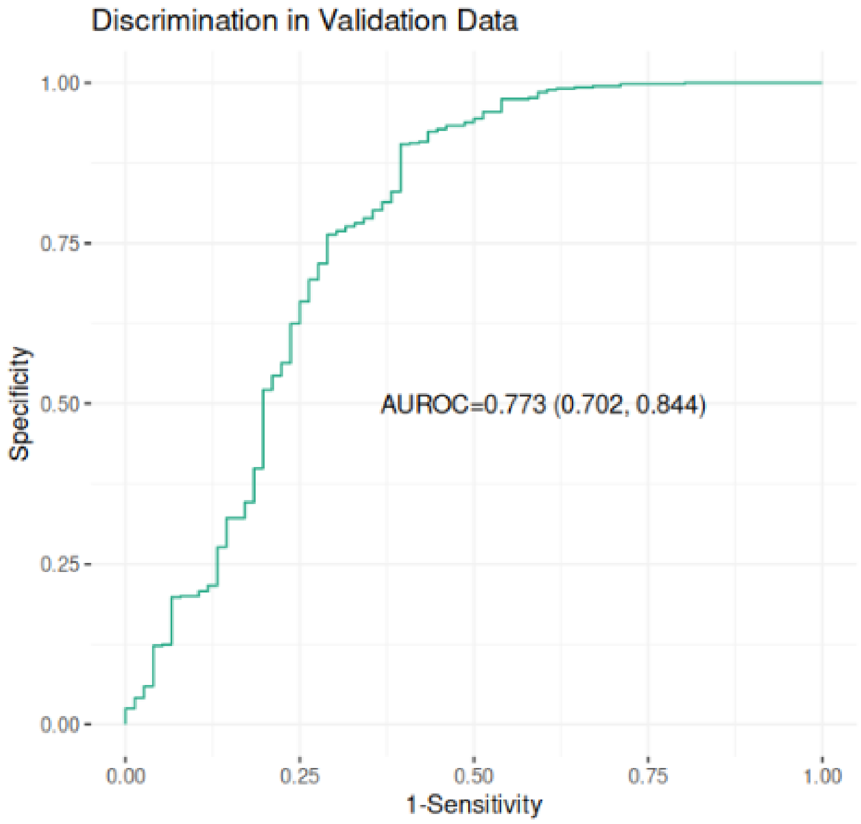

Supplementary Figure S6: Area Under the ROC Curve for predicted probability in the validation data, where Mahalanobis distances were calculated by reference to the distribution of all samples in the validation data.

Supplementary Methods:

Untargeted metabolomics and lipidomics using HRMS

Metabolomics

Ultra Performance Liquid Chromatography (UPLC) was performed on a Shimadzu Nexera-LC system, consisting of a SIL-400 X3 autosampler, DGU-405 degassing unit, SCL-40 system controller, CTO-40C column oven and two LC-40D X3 solvent delivery modules. High Resolution Mass Spectrometry (HRMS) was performed with the ZenoTOF 7600 Mass Spectrometer (AB Sciex, Aus).

C18 runs were performed using a ZORBAX Eclipse Plus C18 column (2.1x100 mm, 1.8-Micron) with mobile phase A consisting of water and mobile phase B consisting of methanol, each containing 0.1% formic acid (v/v). C18 runs were performed over 10-minute intervals with a flow rate of 0.3 ml/min and an initial ratio of 95/5 A/B for 0.2 minutes, before ramping to 30/70 after 5.5 minutes and 5/95 after 6 minutes which was then held for 2 minutes, before an immediate change to 95/5 for the last 2 minutes. The column oven was kept at 40 degrees.

HILIC runs were performed using an Agilent InfinityLab Poroshell 120 HILIC-Z column (2.1x100mm, 2.7-Micron) with mobile phase A consisting of water and mobile phase B consisting of acetonitrile-water (90:10 v/v), each containing 0.1% formic acid (v/v) and 1% ammonium formate (v/v). HILIC runs were performed over 11-minute intervals with a flow rate of 0.3 ml/min and an initial ratio of 2/98 A/B for 1.5 minutes, before ramping to 30/70 after 5 minutes, 50/50 after 6 minutes and 95/5 after 6.5 minutes which was then held for 2 minutes before an immediate change to 2/98 for the last 2.5 minutes. The column oven was kept at 40 degrees.

Metabolomic HRMS was performed using the following instrument settings for a 10-minute method duration. Ion source gases 1 and 2 were set to 45 and 40 psi respectively. Curtain gas was set to 30 psi and CAD gas was set to 7 psi. Source and gas temperature was set to 450 degrees Celsius. Spray voltage was set to 4500 V. TOF MS was performed with masses between 50 and 1000 Da and an accumulation time of 0.25 seconds. A declustering potential of 70 V was used with a spread of 10 V, and collision energy of 10 V with 0 V spread. TOF MSMS was performed with masses between 80 and 1000 Da and an accumulation time of 0.1 seconds. A declustering potential of 70 V was used with a spread of 10 V, and collision energy of 30 V with 10 V spread.

Lipidomics

UPLC was performed on a Shimadzu Nexera-LC system, consisting of an SIL-30 AC autosampler, DGU-20A5R degassing unit, SCL-30 system controller, CTO-30A column oven and 2 LC-30 AD

solvent delivery modules. HRMS was performed with the TripleTOF 5600 Mass Spectrometer (AB Sciex, Australia).

Untargeted lipidomic runs were performed using positive and negative modes with a ZORBAX Eclipse Plus C18 column (2.1x100mm, 1.8-Micron) and two mobile phases. Mobile phase A consisted of methanol-water (3:1, v/v) and mobile phase B consisted of isopropanol, with both containing 1% ammonium formate (v/v). Lipidomic runs were performed over 10-minute intervals with a flow rate of 0.3 ml/min and an initial ratio of 70/30 A/B for 0.2 minutes before ramping to 15/85 after 5.5 minutes and then 5/95 after 5.9 minutes which was held for 2.05 minutes before ramping down for 0.05 minutes to the original 70/30 ratio for the remaining 2 minutes. The column oven was kept at 60 degrees.

Lipidomic HRMS was performed using the following instrument settings for a 10-minute method duration. Ion source gases 1 and 2 were set to 45 and 40 psi respectively. Curtain gas was set to 30 psi and CAD gas was set to 7 psi. Source and gas temperature was set to 450 degrees Celsius. Spray voltage was set to 5500 V. TOF MS was performed with masses between 100 and 2000 Da and an accumulation time of 0.25 seconds. A declustering potential of 70 V was used with a spread of 10 V, and collision energy of 10 V with 0 V spread. TOF MSMS was performed with masses between 70 and 2000 Da and an accumulation time of 0.06 seconds. A declustering potential of 70 V was used with a spread of 10 V, and collision energy of 30 V with 10 V spread.

Supplementary Analysis:

Comparison against internal standards

For analysis against internal standards, analytes were divided into groups based on the MS method that provided the most consistent peak area for respective internal standards. It was found that in negative mode there was little benefit to the C18 column method for the chosen internal standards when compared to positive mode C18 and positive/negative mode HILIC methods. The C18 positive mode method was found to produce the best signal for phenylalanine as well as most carnitines. The remaining carnitines as well as some amino acids were found to be most consistent in HILIC positive mode, whilst other amino and organic acids were favoured in HILIC negative mode.

Of the additional samples included in the validation cohort, a sample with argininosuccinic aciduria (ASL gene) and a sample with autosomal dominant MODY type III were outliers for eleven and thirteen different analytes, respectively. Other samples that were outliers for more than one different analyte include known diagnoses glycogen storage disorder type 3 (2), and haemoglobin H (3) and disorders associated with variants in genes *TFAP2* (3), *SMARCA2* (2), *MUSK* (2), *HBB* (beta thalassaemia) (5), *ATP7A* (Menkes disease) (4), *MYO15A* (3), and *TUBB* (2).

In the lipidomic analyses, four specific analytes were notable. Using 18:1-d7-cholesterol +NH4 as an internal standard for comparison, an analyte identified as Chol Ester(28:5) showed increased area ratio for a case sample with known diagnosis of Zellweger syndrome. The same sample also showed elevated area ratio for an analyte identified as LPC(C26:0) +H using 18:1-d7-LPC +H as an internal standard. A sample with known diagnosis of propionic aciduria had an increased area ratio for an analyte identified as SM(35:1), with 15:0-18:1-d7-PC +H as corresponding internal standard. Lastly, a sample with known diagnosis of trisomy 21 had an increased area ratio between an analyte identified as PC(O-38:5) (PC(P-18:0/20:4)) and 15:0-18:1-d7-PC +H as internal standard, along with two further case samples - one diagnosed with mitochondrial respiratory chain disorders and one sickle cell disease carrier.

Additional biomarkers were also explored - chosen due to being known markers of disease for conditions represented in the discovery cohort. These were analysed qualitatively by peak area on a ZenoTOF 7600 as M+H or M-H depending on whether +ve or -ve ion mode was used. Most additional biomarkers did not provide clear outliers (Supplementary Table 3). A case of nonautoimmune hyperthyroidism was a notable outlier for thyroxine, which was included for hyperthyroidism condition as well as other thyroid-related conditions. Three cases of MCAD stood out as outliers for testosterone, included for icthyosis.

Several additional carnitines were included as markers, with a case of haemophilia A an outlier for 3-hydroxyisovalerylcarnitine. A case of LCHAD was the sole outlier for linoleyl carnitine, as well as one of fifteen outliers for 3-hydroxyoleoylcarnitine which was selected as a known biomarker for LCHAD. Other outliers for 3-hydroxyoleoylcarnitine were cases of Crigler-Najjar syndrome, CACT, SMN1 and 11 samples with no known abnormal condition.

DHEA sulfate and 5Z-tetradecenoic acid both had one clear outlier that was not a diagnosed case sample, whilst for glycoholic acid samples with LCHAD and RASopathy RAF1 were outliers.

For citrulline, one case of glycogen storage disorder 1A deficiency appeared to be an outlier as well as a case with no known condition, whilst lysine had a case of Pearson's syndrome as an outlier. C14:1 was included as a known marker for VLCAD deficiency, with four of the nine samples known to be diagnosed with or carriers of VLCAD showing as outliers.

Glycoholic acid had a case of cardiomyopathy as an outlier, which was also an outlier for glucose-6-phosphate alongside a case of small ectopic lingual thyroid. Samples with diagnosis of MCAD were outliers for sebacic acid and suberic acid, whilst one VLCAD sample was an outlier in 5Z-tetredecanoic acid.

Discovery cohort

The additional 194 outliers observed in this study could have occurred through natural variation between samples and may thus be considered false positives. Interestingly, in the case cohort, 25% of samples presented with at least one of the 24 known biomarker standards as an outlier, while in the cohort of healthy newborns, only 7.4% had an outlier. These observations suggest that biomarkers can often generalise and not only identify cases with

the respective condition, but may also identify other, unrelated conditions, possibly due to a general disturbance of regular metabolic functions.

For example, carnitines are not directly involved in the blood coagulation cascade affected by haemophilia A, and yet two haemophilia A case samples were outliers for multiple carnitines. Similarly, a case sample with DiGeorge syndrome (a chromosomal deletion syndrome) was an outlier for multiple carnitines and amino acids. A cardiomyopathy case sample was also an outlier for several carnitines and amino acids. However, this case was also an extreme pre-term birth which could have had a confounding effect on the outlying metabolites. Finally, two out of 20 cystic fibrosis case samples and nine out of 65 carriers for this autosomal recessive condition were outliers for at least one analyte. It is unlikely these could be considered *CFTR*-specific markers as most *CFTR* samples in the cohort were not outliers.

Validation cohort

The validation cohort had a higher percentage of samples that were outliers for at least one analyte, however there were a disproportionate number of outliers from a few specific amino acids. Some of these amino acid analytes were measured in the discovery set with consistently low peak area values, which may have been disproportionately affected by early issues in discovery data, trending the average number lower. As these issues were not present in the validation data set, the trending of validation data to higher peak area for the amino acids resulted in numerous samples with disproportionately high peak area. Inter-batch variability between different sets is one of the major issues that can arise when analysing a large amount of untargeted metabolomic data.

In lipidomic analysis of the discovery cohort Chol Ester (28:5) was an extreme outlier for a Zellweger syndrome case sample. This syndrome is a peroxisomal disorder that has been associated with intracellular cholesterol transport, resulting in insufficient ciliary cholesterol levels. This could also result in build-up of other cholesterol-related molecules such as Chol Ester(28:5). The same extreme area ratio result was also found for LPC(C26:0). LPC(C26:0) is a known marker for peroxisomal disorders like Zellweger syndrome, including in dried blood spot studies.

The other notable outliers found during lipidomic analysis were SM(35:1) for propionic aciduria and PC(O-38:5) for trisomy 21. The propionic aciduria case sample was also a metabolomic outlier as expected due to propionyl-CoA carboxylase (PCC) deficiency resulting in a build-up of propionyl carnitine. The concentration of SM(35:1), a sphingomyelin phospholipid, could also be affected by the deficiency in PCC, giving rise to elevated area ratio compared to other samples. For the analyte PC(O-38:5), more specifically determined to be PC(P-18:0/20:4), one of two trisomy 21 case samples in the discovery abnormal cohort was a clear outlier. Changes in lipid profiles have been previously noted in patients with trisomy 21.
